# Long-Read-Based Genome Sequences of Pandemic and Environmental Vibrio cholerae Strains

**DOI:** 10.1128/MRA.01574-18

**Published:** 2018-12-13

**Authors:** Noémie Matthey, Natália C. Drebes Dörr, Melanie Blokesch

**Affiliations:** a Laboratory of Molecular Microbiology, Global Health Institute, School of Life Sciences, Ecole Polytechnique Fédérale de Lausanne (EPFL), Lausanne, Switzerland; Loyola University Chicago

## Abstract

The bacterium Vibrio cholerae exhibits two distinct lifestyles, one as an aquatic bacterium and the other as the etiological agent of the pandemic human disease cholera. Here, we report closed genome sequences of two seventh pandemic V. cholerae O1 El Tor strains, A1552 and N16961, and the environmental strain Sa5Y.

## ANNOUNCEMENT

Cholera is one of the oldest diseases known and is still a major burden for people in developing countries (1). The disease is caused by Vibrio cholerae, which also thrives in natural environments (2). Toxigenic strains are characterized by the presence of major virulence factors (3), while marine habitats are often dominated by nontoxigenic strains. Studying those strains helps us to understand pathogen emergence (4–8).

We sequenced three V. cholerae strains (A1552, N16961, and Sa5Y) using whole-genome PacBio sequencing. V. cholerae O1 El Tor (Inaba) strain A1552 (originally named 92A1552 [9]) was isolated by the California health authorities from a traveler returning from South America (10, 11), which links it to the Peruvian outbreak in the 1990s (12–14). First used for research in the Schoolnik laboratory at Stanford University, A1552 was rendered rifampicin resistant (9) and now represents the wild type in most laboratories, including ours. V. cholerae O1 El Tor strain N16961 was the first sequenced strain of this species (15). However, as a recent study suggested an inversion in the initial assembly (16), we resequenced N16961. V. cholerae Sa5Y is a 2004 environmental isolate from California (17).

Genomic DNA was isolated from bacteria cultured in lysogeny broth using a Qiagen genomic DNA buffer set combined with Qiagen 100/G Genomic-tips. Sequencing was performed by the Genomic Technology Facility of the University of Lausanne. DNA samples were sheared in Covaris g-TUBEs to obtain fragments with a mean length of 20 kb. The sheared DNA was used to prepare each library with the PacBio SMRTbell template prep kit 1 (Pacific Biosciences) according to the manufacturer’s recommendations. The resulting library was size selected on a BluePippin system (Sage Science, Inc.) for molecules larger than 15 kb, which excluded smaller plasmids. Each library was sequenced on one single-molecule real-time (SMRT) cell with P6/C4 chemistry and MagBeads on a PacBio RS II system at a movie length of 360 min. Genome assembly was performed using the protocol RS_HGAP_Assembly.3 in SMRT Pipe 2.3.0, and circularization of the genomes was achieved using the Minimus assembler of the AMOS software package 3.1.0 using default parameters (18). The assembled genomes were annotated using Prokka 1.12 (19) (Table 1).

**TABLE 1:** Statistics on genome sequences and assemblies

Feature	A1552	N16961	Sa5Y
GenBank accession no.			
Chromosome 1	CP028894	CP028827	CP028892
Chromosome 2	CP028895	CP028828	CP028893
No. of bases	799,549,317	1,750,962,832	635,540,812
No. of reads	46,861	97,399	35,390
Mean read length (bp)	17,062	17,977	17,958
Total no. of contigs (chromosomes 1 and 2)	2	2	2
Maximum contig length (bp)	3,044,896	3,003,695	2,986,375
*N*_50_ (bp)	3,044,896	3,003,695	2,986,375
Contig length after circularization (bp)			
Chromosome 1	3,015,094	2,975,504	2,955,400
Chromosome 2	1,070,374	1,072,331	1,095,478
Total genome size (bp)	4,085,468	4,047,835	4,050,878
Mean coverage (×)	170	351	133
GC content (%)			
Chromosome 1	47.7	47.7	47.8
Chromosome 2	46.9	46.9	46.8

The stock of the A1552 strain described here was previously passed on to Kemter et al., who deposited it in the German Collection of Microorganisms and Cell Cultures (DSM 106276) concomitantly with the release of its genome sequence (20). To improve upon the automated annotation of this study, we checked the annotated gene names of all coding sequences (CDS) and manually added 1,269 commonly used gene names under “gene”/“gene_synonym” for CDS without/with an automatically assigned gene name. Allué-Guardia et al. also recently released an A1552 genome sequence. However, the absence of the mutation in *rpoB* conferring rifampicin resistance (RpoB[S531F]) and the presence of a streptomycin resistance-causing mutation in *rpsL* (RpsL[K88R]) (21) suggest that this isolate represents a lineage distinct from that of the more commonly used rifampicin-resistant strain A1552 described here.

### Data availability.

The genome sequences have been deposited in NCBI GenBank under the accession numbers CP028894 and CP028895 (A1552), CP028827 and CP028828 (N16961), and CP028892 and CP028893 (Sa5Y). The raw reads are available under SRA numbers SRX4011578, SRX4011577, and SRX4011579.
